# Impact of Proteins on the Uptake, Distribution, and Excretion of Phenolics in the Human Body

**DOI:** 10.3390/nu8120814

**Published:** 2016-12-15

**Authors:** Richard Draijer, Ferdi A. van Dorsten, Yvonne E. Zebregs, Boudewijn Hollebrands, Sonja Peters, Guus S. Duchateau, Christian H. Grün

**Affiliations:** 1 Unilever R&D Vlaardingen, Olivier van Noortlaan 120, Vlaardingen 3133 AT, The Netherlands; Boudewijn.hollebrands@unilever.com (B.H.); Sonja.kaal@unilever.com (S.P.); Guus.duchateau@unilever.com (G.S.D.); Christian.grun@unilever.com (C.H.G.); 2 Eurofins Spinnovation Analytical, Oss 5342 CC, The Netherlands; Ferdi.vandorsten@gmail.com; 3 Eurofins Global Central Laboratory, Breda 4817 PA, The Netherlands; Yvonne.zebregs@gmail.com

**Keywords:** bioavailability, flavonoids, catechins, protein, resveratrol, valerolactones

## Abstract

Polyphenols, a complex group of secondary plant metabolites, including flavonoids and phenolic acids, have been studied in depth for their health-related benefits. The activity of polyphenols may, however, be hampered when consumed together with protein-rich food products, due to the interaction between polyphenols and proteins. To that end we have tested the bioavailability of representatives of a range of polyphenol classes when consumed for five days in different beverage matrices. In a placebo-controlled, randomized, cross-over study, 35 healthy males received either six placebo gelatine capsules consumed with 200 mL of water, six capsules with 800 mg polyphenols derived from red wine and grape extracts, or the same dose of polyphenols incorporated into 200 mL of either pasteurized dairy drink, soy drink (both containing 3.4% proteins) or fruit-flavoured protein-free drink . At the end of the intervention urine and blood was collected and analysed for a broad range of phenolic compounds using Gas Chromatography–Mass Spectrometry (GC-MS), Liquid Chromatography–Multiple Reaction Monitoring–Mass Spectrometry (LC-MRM-MS), and Nuclear Magnetic Resonance (NMR) spectroscopy techniques. The plasma and urine concentrations of the polyphenols identified increased with all formats, including the protein-rich beverages. Compared to capsule ingestion, consumption of polyphenol-rich beverages containing either dairy, soy or no proteins had minor to no effect on the bioavailability and excretion of phenolic compounds in plasma (118% ± 9%) and urine (98% ± 2%). We conclude that intake of polyphenols incorporated in protein-rich drinks does not have a major impact on the bioavailability of a range of different polyphenols and phenolic metabolites.

## 1. Introduction

Polyphenols are plant secondary metabolites characterized by the presence of more than one phenol group per molecule. These compounds are ubiquitous in fruits, vegetables, cereals, chocolate, and beverages, such as tea, coffee, or wine [1]. Epidemiological, clinical, and experimental studies support a role of polyphenols in the prevention of cardiovascular diseases, malignancies, neurodegenerative disorders, and metabolic syndrome, which sparked the discussion whether dietary reference intake values should be defined for these compounds [2,3,4]. Particularly, the polyphenols of red wine have been linked to the ‘French paradox’, referring to the French low mortality rate from ischaemic heart disease, whilst intake of saturated fat is high [5].

Affinity and binding of proteins from different sources to phenolic compounds is a well-known phenomenon [6]. Whether this interaction also impacts the bioavailability of polyphenols is still a matter of debate, and the number of studies in humans is limited. Egert et al. [7] showed negative effects on the bioavailability of gallated catechins for caseinate, milk, and soy proteins, whilst the non-gallated forms were unaffected. Others have described a null-effect of milk on bioavailability for total plasma tea catechins or antioxidant capacity [8,9]. Accordingly, bioavailability of (epi)catechin in a milk chocolate drink was reported not to be substantially affected [10]. Bioavailability of the flavonols quercetin and kaempferol may not or weakly be affected by milk [11]. In contrast, protein-rich soybean flour has been suggested to protect anthocyanins from metabolism, thereby increasing their bioavailability [12].

However, one may question whether investigation of the impact of proteins on polyphenol parent compounds alone is of physiological relevance, considering the very low bioavailability of polyphenols as such [13]. The bulk of the dietary complex polyphenols ends up in the large bowel and the aromatic rings are metabolized by bacteria. The breakdown products consist of smaller and simpler phenolic acids that are absorbed into the human body in much higher quantities than the parent compounds [14,15,16]. Therefore, preferably alongside assessment of the bioavailability of polyphenol parent compounds, the excretion of phenolic metabolites should be taken into account.

Detection of (conjugated) intact polyphenols in plasma, as well as phenolic acids in urine, requires different analytical methods to be applied in a targeted, as well as untargeted, manner. Exposure of an organism to xenobiotics (e.g., polyphenols) results in subtle modifications in biochemical composition of plasma and urine, which can be profiled using ^1^H Nuclear Magnetic Resonance (NMR) spectroscopic analysis [17,18,19]. The approach requires minimal sample preparation, and thus eliminates the necessity of making a priori assumptions as to the relative importance of various metabolite classes. Although ^1^H NMR spectroscopy generates a comprehensive profile of exogenous and endogenous metabolites in biofluids, the sensitivity of this technique is limited to the detection of metabolites present at concentrations higher than approximately 10 μM. Thus, targeted analyses of simple phenolics need to be performed by the more sensitive, but labour-intensive technique Gas Chromatography–Mass Spectrometry (GC-MS). The (conjugated) intact polyphenols and their primary metabolites were analysed by using Liquid Chromatography–Multiple Reaction Monitoring–Mass Spectrometry (LC-MRM-MS).

The present study was set up to determine the impact of dairy and soy proteins, present in a relatively complex beverage format, on the absorption and appearance of polyphenols in the blood circulation as well as the urinary excretion of their phenolic metabolites. To that end, phenolic compounds were measured in blood and urine after consumption of red wine and grape polyphenols formulated in (1) a dairy drink (protein- and casein-rich); (2) a soy drink (protein-rich but casein-free); (3) a fruit-flavoured drink (protein-free) compared to polyphenols incorporated in gelatine capsules taken with water.

## 2. Experimental Section

### 2.1. Study Design

In the present placebo-controlled study, subjects were randomly assigned to one of the treatment sequence of a full crossover Williams design [20]. The following five treatments were allocated to the subjects:

- six cellulose-filled placebo hard-shell gelatine capsules taken with 200 mL of water (control)
- six hard-shell gelatine capsules containing a wine/grape extract mix taken with 200 mL of water (positive control)
- 200 g fruit-flavoured drink containing the wine/grape extract mix
- 200 g dairy drink containing the wine/grape extract mix
- 200 g soy drink containing the wine/grape extract mix

More information about the test products is provided in Section 2.3.

The total duration of the study was five weeks consisting of five consecutive five-day treatment periods with four two-day washout periods (WO) in-between. During each treatment period, subjects daily consumed one of the five test products one hour before breakfast. At the fourth day of each treatment period after intake of the test product subjects started collecting their urine for 24 h. On the fifth day of each period, subjects visited the study facility in a fasted state, handed in their 24 h-pooled urine collection container and received the last test product dose. On this day, blood was collected prior to (*t* = 0) and 1, 2, and 3 h after intake of the test product.

**Figure 1:**
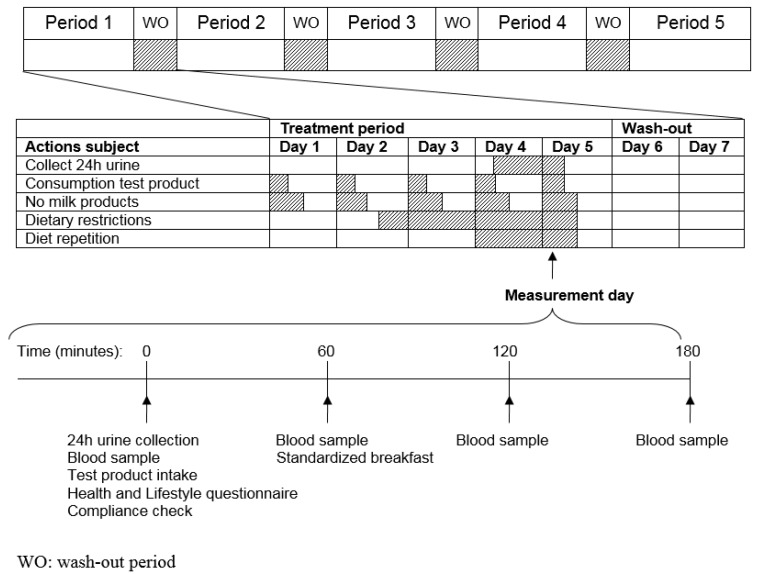
Study overview. In each period the subjects received one of the five treatments. Each period consisted of five days of treatment and two days of washout. On day 5, the measurement day, 24h urine was collected and venous blood was collected prior to (*t* = 0) and 1, 2 and 3 h after intake of the test product.

During the treatment periods subjects had to comply with a number of dietary restrictions. During each treatment period subjects were not allowed to consume milk products in the first three hours after intake of the test product. From day 2 in the evening (10.00 p.m.) until the last blood withdrawal on day 5, subjects were on a low polyphenol diet, avoiding consumption of wine, chocolate, coffee, and tea, and were not allowed to consume alcohol or fish (these food products may interfere with the spectral analysis of plasma and urine samples). The time periods were chosen in such a way that it would bring as little as possible inconvenience to the subjects, but still long enough to likely ensure reaching a steady state. On day 4 of each treatment period, subjects had to repeat the diet that they consumed on day 4 of the first treatment period. An overview of the treatment periods, restrictions, and measurement days is given in Figure 1.

### 2.2. Recruitment of Subjects

#### 2.2.1. Sample Size

The power calculation was based on urinary hippuric acid concentration as general indicator of uptake and excretion of phenolic metabolites. Based on a previous study [15], it was expected that urinary hippuric acid concentrations would increase with 0.35 g per 24 h urine collection with the selected dose of polyphenols in capsules as compared to placebo. A reduction of 25% (0.26 g increase in hippuric acid compared to placebo) due to interference of the food matrix was considered acceptable. In order to detect a significant increase of 0.26 g hippuric acid compared to placebo, with a within-subject variance of 0.11 g, a power of 80%, an alpha of 0.05, thirty subjects were required (Dunnett test correcting for multiple comparisons).

#### 2.2.2. Recruitment and Screening

Apparently healthy males (18–70 years) were recruited among inhabitants of Vlaardingen and surroundings by sending a personal letter to volunteers in a Unilever subject database (1220 letters).

Sixty-eight persons were interested in joining one of the four information meetings. Sixty-one persons completed a questionnaire about general health and wellbeing, which covered a number of inclusion and exclusion criteria. Inclusion criteria were male gender, aged ≥18 and ≤70 years, body mass index (BMI) ≥19 and ≤30 kg/m^2^, reported alcohol consumption <28 alcohol units/week, urinary and plasma clinical chemical parameters within the normal reference range. Exclusion criteria were the habit of smoking during the past year, a recorded history or current metabolic diseases, chronic gastrointestinal disorders, cardiovascular or renal disease, currently on a medically prescribed or slimming diet, reported intense sporting activities >10 h/week or taking prescribed medical treatment possibly interfering with the bioavailability of polyphenol metabolites (e.g., systemic antibiotics). Based on this questionnaire, 47 persons were invited for a second screening visit. Three persons cancelled their appointment for screening and three persons did not show up. On the morning of the screening appointment, all volunteers collected a urine sample and handed it in at the test facility. Forty-one volunteers signed an informed consent form before any measurement was performed. Subsequently, weight and height were measured, the accessibility of the veins was confirmed and 7 mL blood was taken to determine liver enzymes and to measure complete blood count. The morning urine samples were analysed by means of a dipstick. A total of eight subjects were excluded based on the results of the screening, considering the inclusion and exclusion criteria.

#### 2.2.3. Participation

Thirty-three persons started the run-in period. A statistician randomised the personal codes to one of the 10 treatment sequences taking into account as much as possible order and period effects (Williams design). The personal code-test product combinations were kept concealed to investigators and subjects.

Subjects registered compliance to test product intake and background diet in a diary. For each treatment period, subjects received a separate diary. To make it easier for the subjects to repeat the diet on days 4 and 5 in all treatment periods, a copy of their own diary of treatment period 1 was given to each subject. Furthermore, to check compliance to test product intake subjects were requested to return the empty boxes and bottles on the measurement days. The present study has been approved by the independent ethics committee of the University of Wageningen (registration No. 07/07-ABR16833).

### 2.3. Study Test Products

The polyphenol-rich powder consisted of 870 mg red wine extract (Provinols, Seppic, Paris, France) and 540 mg grape juice extract (MegaNatural™ Rubired of Polyphenolics, Madera, CA, USA), containing in total 141 mg anthocyanins, 24 mg flavan-3-ols, 16 mg procyanidins, 10 mg phenolic acids, 9 mg flavonols, and 1 mg stilbenes. This mix was together with 900 mg of micro-crystalline cellulose formulated into six hard-shell gelatine capsules (size 00, Capsugel^®^, Bornem, Belgium). The active and placebo capsules (only containing cellulose) were formulated by Well Plus Trade (Hamburg, Germany). The wine/grape extract mix was also added at the same dose to each serving of the three drinks. The drinks (dairy drink, soy drink, and fruit-flavoured water) were produced in the pilot plant of Unilever Research and Development, (Vlaardingen, The Netherlands). Capsules were stored at room temperature and drinks at −20 °C until one week before distribution to the subjects. After microbiological clearance, subjects received the test products plus one spare product at the instruction meeting and at the measurement day of the previous treatment period. Each subject consumed one dose of six capsules or one bottle (200 mL) per day in a fasted state, one hour before breakfast. The first four days subjects consumed the test products at home. The fifth day they received their final dose at the test facility after the first blood collection. Nutrient composition and pH of the three drinks were determined before and after the intervention (Table 1).

### 2.4. Collection Blood and Urine Samples

On day 4 after discarding their first morning urine, subjects started collecting their urine for a period of 24 h. Urine was collected in suitable containers with metaphosphoric acid as preservative. After measuring the volume of the 24 h pooled urine collection and homogenization, four 10 mL samples were stored at −20 °C as soon as possible. Blood samples were collected on day 5 of each treatment period just before (*t* = 0) and 1, 2, and 3 h after test product intake. On each time point 6 mL of blood was collected by venapuncture from the antecubital vein in tubes containing lithium heparin as anticoagulant. The blood samples were centrifuged directly after withdrawal at 1500× *g*, 10 min, 4 °C. Subsequently plasma samples were aliquoted in three samples of 0.8 mL and stored at −80 °C.

### 2.5. Laboratory Analyses

Polyphenol metabolites were determined in both urine and plasma samples, using different methods of analysis. For urine samples ^1^H-NMR, GC-MS, and LC-MRM-MS data were obtained. Both GC-MS and LC-MRM-MS data were acquired from the plasma samples.

- ^1^H-NMR profiling was used to generate a comprehensive profile of metabolites (both endogenous and exogenous)
- GC-MS was used to produce a fingerprint of phenolic acids in urine
- LC-MRM-MS was used to quantify selected intact and metabolized polyphenols in plasma

#### 2.5.1. Quantitative Determination of Conjugated and Non-Conjugated Polyphenols in Plasma by LC-MRM-MS

All samples of a subject were analysed in one run with two quality control samples (QCs). The QCs were prepared by spiking blank human plasma (Pooled Normal Human Plasma, Innovative Research Inc., Novi, MI, USA) with polyphenol pure standards at a final concentration of 100 ng/mL. To 200 μL plasma, 20 μL of stabilizer solution (10% ascorbic acid containing 0.1% EDTA), 20 μL of 1.5 M NaOAc (pH 4.8), 40 μL of internal standard (±)-taxifolin (250 ng/mL in MeOH/H_2_O 1:1), and 500 units of D-glucuronidase (*Helix pomatia* type H-1 containing sulphatase, Sigma-Aldrich, Zwijndrecht, The Netherlands) was added, mixed, and incubated at 37 °C for 45 min. The reaction was stopped by adding 300 μL of water and 10 μL of 2 mol/L HCl. Polyphenols were isolated by extracting twice with ethyl acetate. Ten microliters (10 μL) of 0.4% ascorbic acid was added to the combined organic layers and dried under N_2_ at room temperature. Samples were dissolved in 100 μL methanol, vortexed, and sonicated for 10 min. One hundred microliters (100 μL) of water was added, vortexed, and sonicated for 10 min. Samples were centrifuged at 17,000× *g* for 10 min. Supernatants were transferred to a Greiner 96-well plate for analysis by LC-MRM-MS.

The LC-MRM-MS system consisted of an Agilent 1200SL binary pump (Agilent Technologies, Amstelveen, The Netherlands) equipped with a thermostated HTC PAL autosampler (CTC Analytics, Zwingen, Switzerland), an Agilent 1200 series degasser, and an Agilent 1200 series column oven connected to an Agilent 6410 triple quadrupole mass spectrometer. Samples were stored in the autosampler tray in the dark at 10 °C. Intact polyphenols and metabolites were separated on an XBridge Phenyl (2.1 × 150 mm, 3.5 μm) (Waters, Etten-Leur, The Netherlands) reversed-phase column protected by a guard column and eluted using a 45-min binary solvent gradient using solvents A (0.1% (*v/v*) acetic acid in MilliQ water) and B (0.1% (*v*/*v*) acetic acid in acetonitrile) as follows: 0–3 min 2% B, 3–4 min 2%–10% B, 4–14 min 10%–20% B, 14–29 min 14%–100% B, 29–34 min 100% B, 34–35 min, 100%–2% B, 35–45 min 2% B. The flow rate was 0.2 mL/min and the column was thermostated at 55 °C. Injection volumes were 5 μL. After every 20 plasma injections, the column was washed with methanol for 30 min. Blank injections preceded plasma injections. The mass spectrometer was operated in negative ion mode using an Agilent electrospray source. Compounds were analysed by multiple-reaction monitoring (MRM). Data were processed using Agilent’s MassHunter Quantitative Analysis software (Agilent Technologies, Amstelveen, The Netherlands).

#### 2.5.2. Semi-Quantitative Analysis of Colonic Metabolites Excreted in Urine by GC-MS

Urine samples were analysed by GC-MS for semi-quantitative determination of gut microbial breakdown products of dietary polyphenols. The protocol for urine sample preparation and subsequent GC-MS analysis has previously been described in detail [21]. Phenolic acids including 3-hydroxyphenylacetic acid, 3-hydroxyhippuric acid, and 4-hydroxyhippuric acid were identified on the basis of a specific retention-time-*m/z* pair, and by comparison with the GC-MS data of authentic reference standards. Semi-quantification of the phenolic acid concentrations was achieved by integration of the characteristic peaks in the total-ion-chromatogram. Peak areas were normalized to the peak area of the internal standard (*trans*-cinnamic acid-*d*_6_), and the volume of the 24 h urine sample was used to arrive at an estimate of the 24-h cumulative excretion per phenolic acid.

#### 2.5.3. Quantification of Hippuric Acid in Urine by NMR Spectroscopy

High-resolution 1D-^1^H-NMR profiling of 24 h urine samples was performed basically as described previously [15]. In brief, NMR samples were prepared by (1:2) mixing of urine with a phosphate buffer solution (pH 6.5), containing 20% D_2_O and 0.05 mg/mL 3-(trimethylsilyl)propionic acid-*d*_4_ sodium salt (TSP) as a chemical shift reference. ^1^H-NMR spectra were acquired at 600.13 MHz and at a temperature of 300 K on a Bruker Avance 600 NMR spectrometer equipped with a 5-mm TXI probe. A standard water-suppressed Noesypresat pulse sequence was used collecting 128 scans with 32 K data points over 8993 Hz. The spectra were manually phase and baseline corrected using Topspin 1.3 software (Bruker Analytik, Rheinstetten, Germany). An exponential window function with a line-broadening factor of 0.3 Hz was applied to the free induction decay prior to Fourier transformation. Hippuric acid levels in urine were determined from the peak integral of its aromatic signal at 7.83 ppm and was expressed in grams excreted over a 24-h period and as molar ratio of hippuric acid/creatinine, adjusting for urine analyte concentrations in case of any incomplete urine collection over 24 h [22].

### 2.6. Statistical Analysis

The statistical analyses were performed using the SAS Software (SAS Institute, Cary, NC, USA, version 9.1) on the per protocol dataset. Descriptive analysis consisted of distribution statistics (number of available observations, mean, standard deviation, and 95% confidence intervals) for continuous data. Differences between the active groups and the placebo were evaluated by means of an analysis of variance. Overall mean effects of the plasma concentrations measured at *t* = 0, 1, 2, and 3 h after polyphenol consumption were statistically analysed. A Dunnett test was performed in order to correct for multiple testing between the active groups and the placebo.

## 3. Results

### 3.1. Baseline Characteristics of the Study Population

Thirty-three subjects started this study. One subject dropped out in treatment period 4 due to medical reasons. This person was not replaced. Data that was collected in treatment periods 1, 2, and 3 was included in the statistical analyses. Thirty-two subjects completed the study. The mean age of the study population was 50.6 ± 17.8 years (ranging from 18 to 69 years) with a BMI of 24.6 ± 2.8 kg·m^−2^.

### 3.2. Adverse Events during the Study

A total of 29 reports of adverse events (AEs) were filed. Of these reports nine AE reports were reported by nine subjects receiving the placebo capsules, seven AE reports by six subjects receiving the active capsules, four AE reports by four subjects receiving the dairy drink, four reports by four subjects receiving the juice and five reports by five subjects receiving the soy drink. Most frequently reported AEs were headache and acute nasopharyngitis. There were no significant differences in frequencies of AEs between actives and placebo. No likely relation between any AE and the test products was found.

### 3.3. Compliance with the Test Product and Background Diet and Lifestyle Restrictions

Compliance with the test products was checked by means of a diary in which subjects daily reported the consumption of the test product and the time of consumption. A dietician evaluated compliance at each measurement day. The self-reported compliance of the intake of the test products was very high: 100% for the placebo capsules, the fruit-flavoured drink, and the soy drink. Two subjects consumed four out of five of the dairy drink and one person consumed four out of five of the positive control capsules. All other subjects consumed 100% of the dairy drink and active capsules. Deviations to background dietary guidelines were registered in the same diary and checked by a dietician. In total 21 urine samples and 28 blood samples were excluded from analysis mainly due to use of antibiotics during the study, medical reasons, coffee consumption, or consumption of proteins from other sources than the test products.

### 3.4. Polyphenols and Metabolites in Plasma and Urine

**Figure 2:**
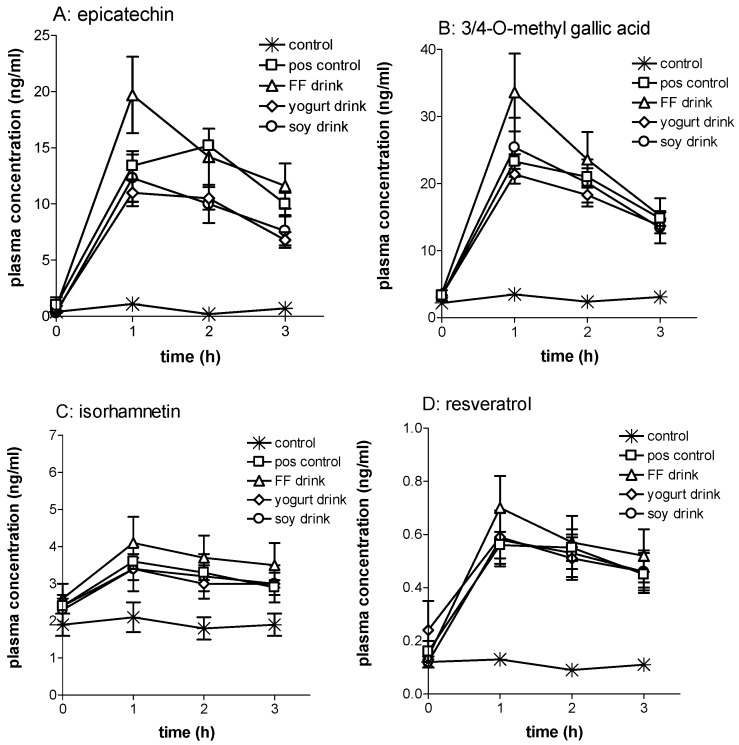
Plasma concentration (mean ± SEM, *n* = 32) versus time profiles for four individual polyphenols (including polyphenol-conjugates) for the five different formulation treatments. (**A**) Epicatechin; (**B**) 3/4-O-methyl gallic acid; (**C**) isorhamnetin; (**D**) resveratrol.

Upon consumption of the polyphenol-rich test products concentrations of phenolic compounds increased by 0.5 to 20-fold within the first three hours after consumption (Table 2; Figure 2). Urinary phenolic acid concentrations increased up to three-fold (Table 3). Urinary excretion of creatinine was not significantly different between interventions and periods (data not shown), indicating good compliance for 24 h urine collection. Compared to capsule ingestion, consumption of polyphenol-rich beverages containing either dairy, soy, or no proteins had minor to no effect on the bioavailability and excretion of phenolic compounds in plasma (mean ± standard error: 118% ± 9%) and urine (98% ± 2%).

## 4. Discussion

The present study demonstrates that the impact of proteins derived from animal and plant sources on the bioavailability of a range of polyphenols and their metabolites is minor to none for the first hours after consumption. Plasma concentrations of a number of identified phenolic compounds increased by approximately ten-fold or more, irrespective of the test product. Resveratrol (conjugates) plasma concentrations increased three to four times unaffected by the protein content, which is in accordance with bioavailability data described previously for resveratrol-containing wine consumed with different meals [23]. All test products increased the plasma concentration of the methylated metabolite of quercetin, isorhamnetin (phase II methyltransferase metabolite), by 50% or more. Previous research indicated that high fat meals may enhance the bioavailability of quercetin, resulting in higher plasma concentrations of isorhamnetin [24], but proteins do not seem to contribute to that effect. Red wine contains considerable amounts of gallic acid and its methylated form appears readily in plasma after wine consumption [25]. We confirmed the large rise in plasma concentration of methyl-gallic acid in the present study, and observed possibly minor effects of proteins present in the test products. The effects of proteins on the bioavailability of catechin and epicatechin seems mixed with a possible enhancement by soy proteins and inhibition by milk proteins. These data contrast to some extent with absence of effect found for proteins on the bioavailability of green tea non-gallated catechins published previously [7]. The valerolactones, microbial metabolites of flavan-3-ols, reach high plasma concentrations, typically five to eight hours after consumption of the polyphenols [14,26]. Nevertheless, these metabolites increased 20-fold within the first three hours after consumption of the test products. Neither soy proteins nor dairy proteins seem to affect the valerolactone plasma concentrations within these first hours. Despite the relatively large portion of anthocyanins present in the test products, we were not able to detect these phenolic compounds. This may reflect the low bioavailability of anthocyanins, as shown by others, reaching at most nanomolar plasma concentrations with similar test dosages of grape-derived anthocyanins as we have included in the present study [27,28].

We assume that the study design has contributed significantly to these clear-cut results. Subjects were on a low polyphenol diet, and had to repeat as much as possible their intake pattern each intervention week. Baseline plasma concentrations of each phenolic compound was, repeatedly, low, enabling us to show increase in phenolic compound plasma concentrations due to the interventions. Moreover, we used a number of state-of-the art measurement tools for identification. What could, however, be considered a weakness in the current study design is the choice of the non-food reference format; gelatine capsules. As such, hard-shell capsules are an attractive format for this type of studies since they can be easily prepared to accommodate study design, extract type, and dose. Ideally, we would have used a non-protein capsule shell material such as hydroxy-propyl-methyl-cellulose (HPMC). This would have created a true non-protein reference and control situation. Unfortunately, HPMC material interacts with polyphenols, limiting severely the release in simulated gastrointestinal (GI) tract condition of the HPMC capsule content [29]. This effect is absent or less pronounced in the case of gelatine capsules, which are designed to disintegrate within minutes in the GI tract. In this study, however, we used six capsules, size 00, consisting in total of 0.75 g gelatine, and if compared to the 6.8 g of protein in the soy and dairy drinks may no longer be negligible. Similarly, the impact of 0.9 g cellulose (filler) in the capsules on the bioavailability of polyphenols is not known. It may explain why the plasma concentrations of the phenolic compounds seem to reach somewhat higher levels with the fruit-flavoured drinks compared to the capsule format (Figure 2).

One should realize that the data in the present study are relevant for consumer products such as milk chocolate drinks, tea, and coffee with milk and fruit drinks containing proteins, but is not conclusive towards complex meals. Different meal compositions may indeed impact the bioavailability of certain polyphenols, albeit may often be difficult to judge which macro- or micronutrients interfered [12,23,24,27,30]. Depending on the type of the polyphenols this impact of a meal on the bioavailability of polyphenols may vary to a large extent with many compound and physiological variables involved, all controlling absorption and bioavailability.

We also found no indication that urinary excretion of a range of phenolic acids combined with either milk or soy proteins was inhibited. This contrasts to the observations published by Urpi-Sarda et al. [31] who investigated phenolic excretion after consumption of cocoa with milk. Milk significantly reduced the urinary excretion of phenolic acids, including hippuric acid and 4-HHA that were unaffected in the present study. Mullen et al. [32] postulated an interesting hypothesis: the excretion of phenolic metabolites may be more affected by (milk) proteins of cocoa products relatively low in polyphenols (70 mg in their study) compared to highly concentrated ones. This may be a plausible explanation for the controversy among studies, although so far the phenolic bioavailability and metabolism of products with low vs. high levels of polyphenols have not been tested head-to-head. Our more precise conclusion may thus be that bioavailability and metabolism of wine and grape polyphenols is not importantly affected by milk or soy proteins when consumed in relatively high dose. The definition of a (sufficiently) high dose requires more research.

## 5. Conclusions

The bioavailability of polyphenols and the excretion of their phenolic metabolites is not significantly affected when polyphenols are consumed in protein-rich soy or dairy drinks compared to polyphenols formulated in capsules.

## Figures and Tables

**Table 1:** Nutrient composition of the three test drinks.

	Product		
	FF Drink	Dairy Drink	Soy Drink
Protein	0.04	3.4	3.4
Carbohydrates	3.9	6.0	4.5
Fat	1.73	1.40	2.10
Ash	0.04	0.56	0.42
Moisture	94.3	87.3	88.1
Glucose	0.2	0.7	0.1
Fructose	0.1	0.1	0.1
Lactose	<0.05	1.7	<0.05
Sucrose	3.0	2.9	3.4
Maltose	<0.05	<0.05	0.4
pH	4.3	4.0	4.2

Values expressed as g/100 g product. FF drink: fruit-flavoured drink. Ash: residue of inorganic material.

**Table 2:** Peak plasma concentration of sum of free and conjugated polyphenols and metabolites after consumption of different test products (*n* = 32).

	Control	Positive Control	Dairy Drink	Soy Drink	FF Drink
Resveratrol	0.17	0.56 ***	0.61 ***	0.58 ***	0.70 ***
	(0.03–0.30)	(0.43–0.69)	(0.48–0.74)	(0.46–0.71)	(0.57–0.83)
Epicatechin	1.5	15.7 *	12.0 *	22.6 **	20.5 **
	(−6.8–9.8)	(8.4–23.0)	(4.6–19.4)	(15.4–29.8)	(13.3–27.7)
Catechin	0.47	4.4	3.3	12.1 *	5.9
	(−5.2–6.2)	(−0.5–9.3)	(−1.7–8.3)	(7.3–17.0)	(1.0–10.7)
Valerolactone	0.61	13.7 *	12.5 *	12.2	11.6
	(−3.3–4.5)	(10.2–17.3)	(9.0–16.0)	(8.8–15.6)	(8.1–15.1)
M-valerolactone	0.17	2.4 *	3.5 *	3.3 *	3.1 *
	(−0.60–0.94)	(1.7–3.2)	(2.7–4.2)	(2.5–4.0)	(2.4–3.8)
M-gallic acid	3.6	23.5 ***	21.7 ***	25.2 ***	34.1 ***^,#^
	(1.7–5.5)	(21.6–25.3)	(19.8–23.6)	(23.3–27.1)	(32.3–36.1)
Isorhamnetin	2.1	3.7 ***	3.3 ***	3.4 ***	3.9 ***
	(1.6–2.5)	(3.2–4.1)	(2.9–3.8)	(2.9–3.8)	(3.5–4.4)

Mean (95% confidence interval) in ng/mL. Test products were cellulose-filled capsules (control), and wine and grape polyphenols incorporated into either capsules (positive control), a dairy drink, a soy drink, or a fruit-flavoured (FF) drink. Valerolactone: 5-(3′,4′-dihydroxyphenyl)-γ-valerolactone; M-valerolactone: 5-(3′-methoxy-4′-hydroxyphenyl)-γ-valerolactone; M-gallic acid: 3/4-*O*-methyl gallic acid. Statistically significant compared to control (placebo capsules): * *p* < 0.05, ** *p* < 0.001, *** *p* < 0.0001. Statistically significant compared to positive control (polyphenol-filled capsules): ^#^ *p* < 0.0001.

**Table 3:** Relative 24-h cumulative urinary excretion of phenolic metabolites after consumption of different test products with control as the reference (1.0).

	Control	Positive Control	Dairy Drink	Soy Drink	FF Drink
3-HHA	1.0	2.9 ***	2.6 **	3.2 ***	2.6 **
	(0.6–1.4)	(2.2–3.7)	(1.8–3.4)	(2.0–4.5)	(1.8–3.3)
4-HHA	1.0	1.6 *	1.5 *	1.5 *	1.6 *
	(0.7–1.3)	(1.2–1.9)	(0.9–2.0)	(1.0–2.0)	(1.2–2.0)
pyrogallol	1.0	1.3 *	1.1	1.5 ***	1.2
	(0.8–1.2)	(1.1–1.5)	(1.0–1.3)	(1.2–1.8)	(1.0–1.4)
3-HPAA	1.0	2.5 ***	2.4 ***	2.5 ***	2.6 ***
	(0.7–1.3)	(1.9–3.1)	(1.7–3.0)	(1.9–3.0)	(1.9–3.3)
3-HPPA	1.0	1.3 *	1.3	1.4 *	1.3 *
	(0.8–1.2)	(0.9–1.7)	(0.8–1.7)	(0.9–1.9)	(0.8–1.9)
Homovanillic acid	1.0	1.3 *	1.2	1.2 *	1.2 *
	(0.9–1.1)	(1.0–1.5)	(1.0–1.4)	(1.0–1.3)	(1.0–1.4)
Hydrocaffeic acid	1.0	1.4 *	1.1	1.2	1.5 *
	(0.5–1.5)	(1.1–1.7)	(0.8-1.5)	(0.9–1.5)	(1.0–2.1)
Syringic acid	1.0	1.0	1.1	1.1	1.1
	(0.8–1.2)	(0.8–1.2)	(0.8–1.3)	(0.9–1.3)	(0.8–1.3)
Vanilmandelic acid	1.0	1.1 *	1.1	1.1	1.1
	(0.9–1.1)	(1.0–1.3)	(0.9–1.2)	(0.9–1.2)	(0.9–1.2)
Hippuric acid	0.30	0.42 ***	0.38 *	0.42 **	0.39 *
	(0.24–0.35)	(0.37–0.47)	(0.33–0.42)	(0.37–0.48)	(0.32–0.46)
Hippuric acid/creatinine	0.083	0.114 ***	0.108 **	0.116 ***	0.108 **
	(0.070–0.096)	(0.102–0.127)	(0.095–0.120)	(0.104–0.129)	(0.095–0.120)

Mean (95% confidence interval) data for 24-h cumulative excretion of urinary metabolites are shown as relative to control values, except for hippuric acid (in grams) and the hippuric acid/creatinine (in molar ratio). Test products were cellulose-filled capsules (control), and wine and grape polyphenols incorporated into either capsules (positive control), a dairy drink, a soy drink, or a fruit-flavoured (FF) drink. Statistically significant compared to control (placebo capsules): * *p* < 0.05, ** *p* < 0.001, *** *p* < 0.0001. 3-HHA: 3-hydroxy hippuric acid; 4-HHA: 4-hydroxy hippuric acid; pyrogallol: 1,2,3-trihydroxy-benzene; 3-HPAA: 3-hydroxy-phenyl acetic acid; 3-HPPA: 3-hydroxy-phenyl propionic acid.
